# Emotional Profiles and Their Relationship with the Use of Artificial Intelligence in University Students

**DOI:** 10.3390/bs15111573

**Published:** 2025-11-17

**Authors:** Raquel Suriá-Martínez, Fernando García-Castillo, Carmen López-Sánchez, José A. García del Castillo

**Affiliations:** 1 Department of Communication and Social Psychology, University of Alicante, 03690 Alicante, Spain; mc.lopez@ua.es; 2 Department of General Didactics and Specific Didactics, University of Alicante, 03690 Alicante, Spain; fgarciadelcastillo@ua.es; 3 Department of Health Psychology, University of Miguel Hernandez, 03202 Elche, Spain; jagr@umh.es

**Keywords:** artificial intelligence, emotional intelligence, motivational profiles, higher education, academic learning

## Abstract

This study analyzes the relationship between emotional intelligence (EI) profiles and the use of artificial intelligence (AI) among university students, considering its use as an academic, informational, and emotional support resource. It also explores whether there are statistically significant differences between the identified EI profiles and the purposes for which AI is used. Finally, it examines the association between EI and AI use. A total of 352 students from the University of Alicante participated (184 women, 168 men; mean age = 21.4, SD = 2.3). EI was assessed using the TMMS-24 scale (Attention, Clarity, and Emotional Repair). To evaluate AI use, a 12-item ad hoc questionnaire was developed and validated, comprising three dimensions: educational support, informational support, and emotional support. Cluster analysis identified three EI profiles: (1) high and balanced EI, with high scores across all three dimensions; (2) regulatory EI, characterized by moderate attention and high emotional understanding and regulation; (3) repair-deficit EI, showing difficulties in emotional regulation despite moderate perception and understanding. ANCOVA analyses assessed differences between profiles, showing that students with high and balanced EI perceived greater usefulness of AI for educational and informational support, as well as greater emotional support benefits, compared to other profiles. Finally, positive correlations were found between EI and AI use across all three types of support. These findings suggest that EI influences AI use in differentiated ways, highlighting its role as a facilitator of learning, information management, and emotional well-being in higher education.

## 1. Introduction

The modern university operates in an environment characterized by profound transformations. Globalization, digitalization, and, in particular, the emergence of artificial intelligence (AI) have significantly altered teaching and learning processes (González-Campos et al., 2024; Yánez-Jara et al., 2019). In this context, students are required not only to develop technical and disciplinary competencies but also to strengthen emotional and adaptive abilities that enable them to face uncertainty and take advantage of the opportunities provided by emerging technologies (Paik et al., 2025; Salas Román et al., 2022).

In this regard, artificial intelligence emerges as a versatile resource for university students, as it is applicable in academic purposes and can contribute to a more holistic learning experience by combining knowledge development with effective information management and students’ psychological well-being. Thus, AI positions itself as a tool with the potential to strengthen both the cognitive and socio-emotional competencies necessary for academic and personal success (Bartz et al., 2023; Chacon-Chamorro et al., 2024; Rehman et al., 2021).

One of the most studied and relevant socio-emotional competencies in the academic field is emotional intelligence (EI). It is defined as the ability to recognize, understand, and regulate one’s own emotions, as well as to interpret and appropriately respond to the emotions of others. The development of EI is essential in an educational environment increasingly mediated by intelligent technologies. The use of artificial intelligence (AI)-based tools requires not only cognitive abilities but also balanced emotional management and ethical reflection on their application.

Emotional intelligence enables individuals to maintain the motivation, self-regulation, and self-confidence necessary to interact critically and autonomously with AI. However, while AI can offer cognitive and emotional assistance, its effectiveness largely depends on the user’s emotional competencies, which allow for accurate interpretation of technological feedback, sustained motivation, and a balanced interaction between human autonomy and digital dependency. In this regard, EI becomes an indispensable competency for leveraging the benefits of AI without compromising critical thinking, self-awareness, or interpersonal connection (Goleman, 1995; Morales & López-Zafra, 2009).

The construct of EI was initially proposed by Mayer and Salovey (1997), who defined it as a set of abilities aimed at perceiving, understanding, regulating, and effectively using emotions. In the current literature, two main approaches to EI are distinguished: the trait model, which views EI as relatively stable emotional dispositions, and the ability model, which conceptualizes it as a set of developable cognitive skills. Contrasting Goleman’s perspective, focused on applied emotional competencies, with Mayer and Salovey’s approach, centered on measurable abilities, allows for a deeper understanding of how recent developments in EI assessment have advanced toward practical educational applications. These advances integrate self-report scales, situational evaluations, and analyses of EI’s impact on motivation, self-regulation, and collaborative learning.

Although various instruments exist to evaluate emotional intelligence (EI), one of the most widely used is the Trait Meta-Mood Scale (TMMS), a self-report questionnaire developed by Salovey et al. (1995). This instrument identifies three main dimensions of EI: (a) attention to emotions, (b) emotional clarity, and (c) repair of negative emotional states. These dimensions facilitate self-awareness, emotional regulation, and conflict resolution, directly influencing students’ ability to manage academic and technological complexity, including the integration of AI into their learning processes. Attention enables individuals to perceive and reflect on their own emotions; clarity enhances the understanding of personal emotional states; and repair supports the regulation of negative feelings, contributing to better stress management and improved interpersonal relationships (Buenrostro-Guerrero et al., 2012; Martins et al., 2010; Extremera Pacheco & Fernández Berrocal, 2005; MacCann et al., 2020; Sánchez-Camacho & Grane, 2022).

In the university context, studies using the TMMS have shown that the dimensions of clarity and repair are linked to higher empathy levels, greater life satisfaction, and better-quality social relationships (Extremera Pacheco & Fernández Berrocal, 2005; Fragoso-Luzuriaga, 2015). Likewise, students with higher EI levels demonstrate better stress management, stronger intrinsic motivation, and improved interpersonal relationships, all of which positively influence academic performance (Extremera Pacheco & Fernández Berrocal, 2005; MacCann et al., 2020). Moreover, EI enables greater flexibility in facing the demands of a university environment marked by complexity, competitiveness, and constant technological innovation.

From the perspective of technology acceptance models, the integration of Emotional Intelligence (EI) can be understood through the Technology Acceptance Model (TAM; Davis, 1989) and the Unified Theory of Acceptance and Use of Technology (UTAUT; Venkatesh et al., 2003). According to TAM, perceived usefulness and ease of use determine the intention to employ a given technology. In this sense, students with high EI perceive AI as more useful and manageable due to their ability to self-regulate emotions, manage frustration, and maintain motivation, which in turn enhances the strategic adoption of AI. Complementarily, UTAUT proposes that performance expectancy, social influence, and facilitating conditions are moderated by EI; emotionally competent students are more resilient and adaptive, capable of leveraging the educational environment—including tutors, peers, and digital platforms—thus reinforcing the integration of AI into their learning (Selwyn, 2023).

Focusing on the possible forms of support that this technological resource can offer, the recent literature identifies three primary ways AI functions as a source of assistance in higher education. For example, AI can act as an instructional tool, providing personalized tutoring, immediate feedback, and optimization of teaching processes, which allows students to access adaptive learning experiences tailored to their needs. At the same time, AI can serve an informational role, facilitating the search, organization, and synthesis of large volumes of data, thereby improving efficiency in solving complex tasks and projects (Perezchica-Vega et al., 2024). Finally, AI can function as an emotional or generative companion, supporting self-regulation, planning, and academic stress management, as well as promoting creativity and collaboration through the generation of educational resources (Selwyn, 2023).

Understanding these different types of support makes it possible to identify how each interacts with students’ socio-emotional competencies, particularly EI. For instance, students with higher levels of self-awareness and emotional regulation may benefit more from AI as an emotional companion, while those with greater motivation and social skills might take greater advantage of generative tools in collaborative activities (Extremera Pacheco & Fernández Berrocal, 2005; MacCann et al., 2020; Rehman et al., 2021).

Recent research supports these associations. For example, Villafuerte (2024) found that students with high EI report more diverse and strategic uses of AI in academic contexts, whereas those with deficits in emotional repair show more limited interactions and reduced socio-emotional benefits from these tools. Other studies in higher education have confirmed that EI functions as a mediator between motivation, well-being, and technological adoption, supporting both academic performance and self-regulation (MacCann et al., 2020; Sánchez-Álvarez et al., 2020). In this regard, there is broad scientific consensus that EI plays a crucial role in motivation and academic achievement, and that its relationship with AI in emotional assessment represents an emerging area of research (Ahsqui & Martínez, 2024; Cejudo & López, 2017; López De La Cruz & Baldeón-Canchaya, 2024).

However, the use of AI in university contexts also presents significant ethical and technical challenges. These include algorithmic bias, which may perpetuate inequalities; data privacy concerns, which require protection against risks of exposure and misuse; dependency on generative AI, which could limit the development of critical thinking skills if used indiscriminately; and the risk of employing AI as an emotional support tool, as it could replace professional intervention when specialized psychological care is needed. These challenges demand a critical and reflective approach that integrates ethics, transparency, and the development of socio-emotional competencies that complement technology, promoting a balanced and conscious use of these tools (Selwyn, 2023).

The review of existing literature shows that there are still few studies explicitly addressing the relationship between emotional intelligence (EI) and the use of artificial intelligence (AI) in educational contexts, particularly in higher education (Cevallos et al., 2024; López De La Cruz & Baldeón-Canchaya, 2024; Ahsqui & Martínez, 2024). More specifically, no publications have been found analyzing how EI dimensions may condition the frequency, motivation, or purpose of students’ use of AI tools for academic, informational, or emotional goals. In light of this gap, fundamental questions arise: Can EI influence the degree of AI use?

Studying EI profiles alongside the use of AI provides significant theoretical and practical value, as it allows for an understanding of how self-awareness, self-regulation, motivation, and social skills influence the way students perceive, use, and benefit from AI (MacCann et al., 2020; Rehman et al., 2021). Likewise, exploring how the dimensions of Attention, Clarity, and Repair shape the motives for using AI not only helps close an important gap in university research but also contributes to the design of personalized educational interventions, the early detection of emotional risks, and the promotion of more conscious, ethical, and adaptive learning.

In this sense, the present research fits within an emerging field that places the relationship between EI and AI in higher education as a subject of growing scientific and pedagogical interest (Cejudo & López, 2017; Rehman et al., 2021). Therefore, the general objective is to analyze the relationship between university students’ EI profiles and their use of AI in education, considering its impact on academic, informational, and emotional domains.

From this general objective, the following specific objectives are derived: (1) to identify and describe university students’ EI profiles based on the relevance of its dimensions; (2) to determine whether these profiles are related to AI use as a resource for educational support and learning enhancement; (3) to analyze whether the development of emotional intelligence (EI) in university students is associated with the diversity and purpose of AI use, considering its role as a potential resource for promoting emotional well-being, affective regulation, and academic adaptation.

From these objectives, several research hypotheses emerge, among them:

**H1:**  *EI profiles, configured from the combination of intrapersonal and interpersonal competencies, will show significant differences in how students integrate AI into their academic and daily experiences. This hypothesis is supported by the fact that EI has been consolidated as a key element in promoting adaptation, personal well-being, and the quality of interactions in educational environments (Goleman, 1995; Mayer & Salovey, 1997).*

**H2:**  *Emotional intelligence (EI) profiles influence the use of artificial intelligence (AI) in higher education differently, both for educational, informational, and emotional support. This hypothesis is based on the premise that EI profiles, by influencing motivation, self-regulation, and adaptation to new technologies, condition how students use AI as a resource for educational support and learning enhancement (Morales & López-Zafra, 2009).*

**H3:**  *Students with higher levels of emotional intelligence, in terms of attention, clarity, and emotional regulation, tend to use artificial intelligence in a more diverse and balanced manner, which contributes to their well-being, resilience, and improved academic performance.*

## 2. Method

### 2.1. Participants

The sample consisted of 352 students from the University of Alicante, selected through convenience sampling, with ages ranging from 18 to 28 years and a mean age of 21.4 years (SD = 2.3).

Of the total, 178 were women (50.6%) and 174 men (49.4%), distributed across various fields of study: Engineering (n = 85), Education (n = 69), Business Administration (n = 61), Health Sciences (n = 70), and Social Sciences (n = 67).

To assess the homogeneity of gender distribution across academic fields, a Chi-square test of homogeneity was applied. The results indicated no significant differences between men and women according to degree program (χ^2^ = 0.279, df = 4, *p* = 0.991). The expected values under the assumption of uniform distribution were approximately 43 women and 42 men in Engineering, 34 and 35 in Education, 31 and 30 in Business Administration, 35 and 35 in Health Sciences, and 34 and 33 in Social Sciences, thus confirming the homogeneity of the sample.

### 2.2. Instruments

To measure perceived emotional intelligence, a self-report instrument was used: the Trait Meta-Mood Scale-24 (TMMS-24) developed by Fernández-Berrocal et al. (2004). This scale is a shortened version adapted into Spanish from the original Trait Meta-Mood Scale (TMMS) by Salovey et al. (1995), derived from the TMMS-48.

The TMMS-24 consists of 24 items, assessed using a 5-point Likert scale (1 = strongly disagree; 5 = strongly agree), and is organized into three dimensions with 8 items each: Attention to feelings, Emotional clarity, and Repair of negative emotions.

This instrument was chosen for its ease of application and its validation in both young and adult populations (Fernández-Berrocal et al., 2004). The Spanish version of the scale has demonstrated internal consistency indices above 0.80 (Attention, α = 0.84; Clarity, α = 0.82; Repair, α = 0.81). In the present study, reliability was α = 0.83 for Attention, α = 0.81 for Emotional Clarity, and α = 0.80 for Emotional Repair.

To evaluate university students’ use of artificial intelligence (AI), an ad hoc questionnaire was developed focusing on three dimensions: educational support, informational support, and emotional support. The instrument consisted of 12 items formulated as statements, using a 5-point Likert scale (1 = strongly disagree; 5 = strongly agree). The first dimension, *educational support*, included 2 items that explored students’ perceptions of AI usefulness for understanding content, organizing and planning study, generating ideas, solving academic problems, and fostering autonomous learning. The second dimension, *informational support*, also containing 7 items, assessed AI’s ability to provide reliable information, facilitate rapid access to complex concepts, support academic decision-making, and complement traditional study materials. The third dimension, *emotional support*, likewise comprised four items, examining how AI can reduce anxiety, provide guidance in academic difficulties, offer a sense of companionship during study, and increase motivation and concentration on academic goals.

Additionally, the questionnaire included an optional open-ended comments section to collect further experiences regarding students’ interaction with AI.

Regarding validity, the questionnaire was designed based on an exhaustive review of literature on AI tools in educational contexts and on academic and emotional support in university students. Item relevance and clarity were evaluated by a panel of education and psychology experts, ensuring content validity. A subsequent exploratory factor analysis (EFA) confirmed that the proposed three-factor structure aligned with the theoretical dimensions, explaining approximately 70% of total variance: 26% for educational support, 24% for informational support, and 18% for emotional support (see Table 1).

To ensure construct validity, data adequacy tests were conducted prior to the EFA. The Kaiser-Meyer-Olkin (KMO) index yielded a value of 0.84, indicating sampling adequacy for factor extraction. Additionally, Bartlett’s test of sphericity was significant (χ^2^(66) = 512.37, *p* < 0.001), confirming sufficient correlations among items to justify factor analysis.

**Figure 1:**
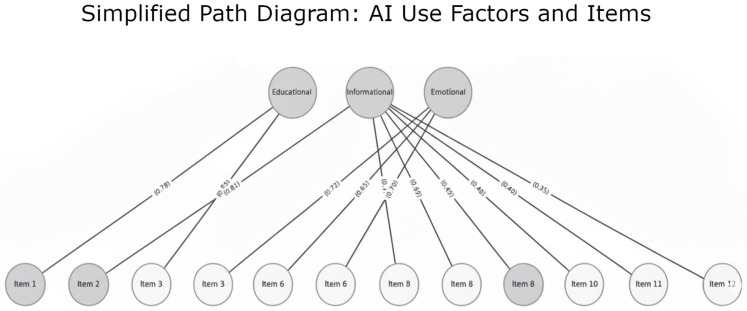
Path Diagram of the Three-Factor Mode. The high-resolution diagram can be found at the end of the text.

The EFA was performed using the factor extraction method with varimax rotation, identifying a three-factor structure consistent with the theoretical dimensions: educational, informational, and emotional support. The results showed that the first factor, educational support, explained 26% of the variance; the second, informational support, 24%; and the third, emotional support, 18%, accounting for a total of 70% explained variance. Factor loadings ranged between 0.45 and 0.82, indicating that all items adequately associated with their respective factors. To further support construct validity, a confirmatory factor analysis (CFA) was conducted. The results indicated that the three-factor model demonstrated a good fit, with the following indices: CFI = 0.96, TLI = 0.95, RMSEA = 0.045 (90% CI: 0.032–0.058), and SRMR = 0.038. All standardized factor loadings were significant (*p* < 0.001) and exceeded 0.50, confirming that each item reliably represented its intended theoretical dimension (see Figure 1).

The reliability of the instrument was evaluated using Cronbach’s alpha, yielding satisfactory values for each dimension: α = 0.85 for educational support, α = 0.82 for informational support, and α = 0.80 for emotional support. The overall reliability coefficient was α = 0.87, indicating adequate internal consistency.

### 2.3. Procedure

Data collection was carried out through the administration of a questionnaire to students from different degree programs at the University of Alicante. First, authorization was requested by the Vice-Rectorate of Students to disseminate the study and invite voluntary participation. Subsequently, an online announcement was posted on the university’s virtual campus, outlining the study objectives and inviting students to participate voluntarily.

The questionnaire was hosted on a web platform, and students accessed it through the link provided in the announcement. Completion of the questionnaire took approximately 10 min. Before answering, informed consent was requested, ensuring participants understood the study’s objectives, procedures, data confidentiality, and the voluntary nature of their participation, in accordance with ethical standards for research with university populations.

### 2.4. Data Analysis

With the aim of identifying distinct emotional intelligence (EI) profiles, a cluster analysis was conducted combining hierarchical and non-hierarchical approaches. In the first phase, an agglomerative hierarchical method was applied using Euclidean distance as a similarity measure and average linkage as the clustering criterion. This procedure allowed for the exploration of the underlying data structure and the determination of the optimal number of clusters based on scores in the three EI dimensions measured by the TMMS-24 (Attention to Feelings, Emotional Clarity, and Emotional Repair). Various clustering quality metrics were calculated to evaluate the validity of the resulting structure. The results indicated that a three-cluster solution provided an adequate balance between internal cohesion and external separation. Specifically, the average silhouette index was 0.482, suggesting a clearly differentiated cluster structure.

To complement this analysis, Gaussian mixture models (GMM) were fitted to compare model fit using the AIC and BIC information criteria. The lowest values for both indicators were obtained for the three-cluster solution, supporting its selection as the most parsimonious and statistically robust structure.

This Table 2 presents the average silhouette index obtained from K-means clustering and the AIC and BIC values from the GMM analysis for different cluster solutions. The results support the choice of a three-cluster solution as providing the optimal balance of cluster cohesion, separation, and overall model fit.

**Figure 2:**
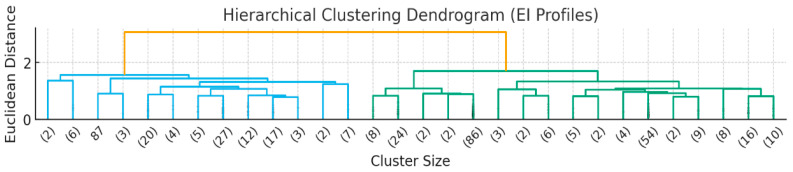
Hierarchical Clustering Dendrogram.

The convergence between the validation metrics and the theoretical consistency of the results with previous research on EI profiles in university populations (Figure 2) reinforced the relevance of this solution. Visual inspection of the hierarchical dendrogram (Figure 2) and analysis of the agglomerative coefficients showed a substantial increase in distance when merging three and two clusters, confirming that the three-cluster solution adequately represented the heterogeneity of the sample.

In the second phase, the K-means method was applied with three clusters, using the centroids obtained from the hierarchical analysis as initial seeds. This procedure allowed for optimization of the final partition and yielded a stable classification of participants. The means and standard deviations of each EI dimension by cluster are presented in Table 3.

Next, to assess whether different EI profiles influenced perceived use of AI in its three dimensions (educational, informational, and emotional support), analyses of covariance (ANCOVA) were conducted, considering gender and age as covariates to control for their possible effects on differences among profiles. Post hoc comparisons were performed using the Bonferroni method to detect significant differences between clusters, and Cohen’s *d* effect size was calculated to quantify the magnitude of differences.

Values of F, *p*, and partial η^2^ were reported to assess statistical significance and global effect size, as well as means and standard deviations for each cluster across the three AI use dimensions. Significance was set at *p* < 0.05. This approach allowed for the determination of how different emotional intelligence profiles relate to the perception and use of AI in university contexts, providing evidence on the influence of emotional competencies in the adoption of educational and emotional support technologies.

To address Objective 3, which aimed to examine whether the dimensions of emotional intelligence (EI) are associated with types of artificial intelligence (AI) use, both correlational and regression analyses were conducted. First, Pearson correlation coefficients were calculated between overall and dimension-specific AI scores and the EI dimensions, in order to assess the strength and direction of the linear relationships between emotional competencies and perceived AI use.

Next, multiple linear regression analyses were performed, with the three EI dimensions entered as predictor variables and each dimension of AI use treated as a dependent variable. This approach allowed for the identification of which emotional intelligence profiles significantly account for the different types of AI support utilized by students.

All analyses were conducted using IBM SPSS Statistics (version 23.0), with the significance level set at *p* < 0.05.

## 3. Results

### 3.1. Emotional Intelligence Profiles

**Figure 3:**
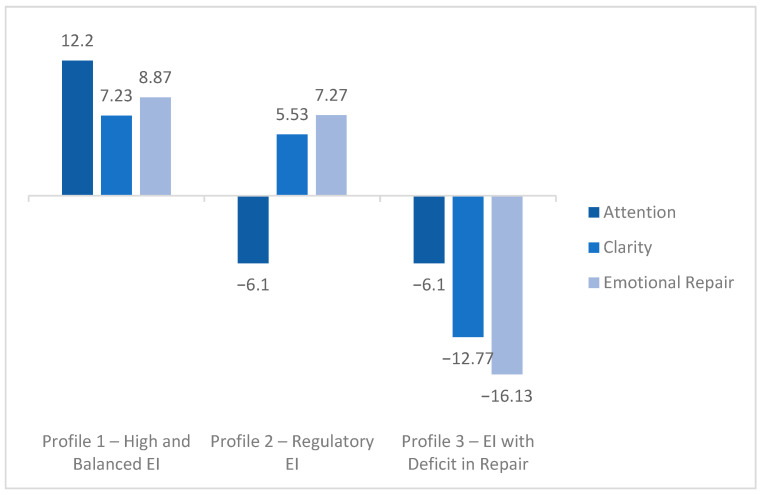
Emotional Intelligence Profiles.

The cluster analysis based on TMMS-24 dimensions identified three EI profiles among the 352 university students:

The Profile 1—High and balanced EI (40%, n = 141): High scores in Attention (77.5%), Clarity (80%), and Emotional Repair (77.5%). This group demonstrates comprehensive emotional development, with the ability to perceive, understand, and manage emotions effectively. The Profile 2—Regulatory EI (35%, n = 123): Moderate Attention (50%) but high Clarity (77.5%) and Emotional Repair (75%). Students in this profile understand and regulate their emotions effectively without focusing excessively on each emotion, supporting decision-making and resilience to academic stress. The Profile 3—EI with deficit in Emotional Repair (25%, n = 88): Moderate Attention (50%) and Clarity (50%), but low Repair (40%). This pattern suggests that students partially perceive and understand their emotions but struggle to regulate them adequately, which may affect stress management and academic motivation (see Figure 3).

### 3.2. Use of AI According to EI Profiles

The results showed significant differences in perceived AI use across the three emotional intelligence (EI) profiles.

Regarding educational support, students with a high and balanced emotional intelligence profile (Profile 1) reported the highest mean score (M = 4.20, SD = 0.48), followed by those with regulatory emotional intelligence (Profile 2) (M = 3.90, SD = 0.52), and finally, students with an emotional repair deficit (Profile 3) (M = 3.50, SD = 0.55). ANOVA analysis revealed significant differences between profiles, F(2, 349) = 40.10, *p* < 0.001, with a large effect size (partial η^2^ = 0.18), indicating that students with higher emotional development perceive artificial intelligence as a more useful tool for organizing, planning, and enhancing academic learning.

A similar pattern was observed for informational support. Profile 1 students had a mean of 4.10 (SD = 0.50), Profile 2 students 3.85 (SD = 0.55), and Profile 3 students 3.45 (SD = 0.54). ANOVA showed significant differences, F(2, 349) = 36.50, *p* < 0.001, with a moderate-to-high effect size (partial η^2^ = 0.17). This suggests that students with higher emotional intelligence use AI more effectively for searching, organizing, and understanding academic information.

Regarding emotional support, Profile 1 students scored a mean of 3.80 (SD = 0.60), Profile 2 students 3.60 (SD = 0.62), and Profile 3 students 2.90 (SD = 0.65). Differences were significant, F(2, 349) = 35.20, *p* < 0.001, with a considerable effect size (partial η^2^ = 0.16), indicating that emotional regulation directly influences the perception of AI as a resource for stress management, self-regulation, and academic motivation.

Post hoc analyses using the Bonferroni correction confirmed that all three emotional intelligence profiles differed significantly across all dimensions of AI use. In terms of educational support, Profile 1 scored significantly higher than both Profile 2 (*p* < 0.01) and Profile 3 (*p* < 0.001), while Profile 2 also outperformed Profile 3 (*p* < 0.01). A similar pattern emerged for informational support, with Profile 1 achieving higher scores than Profile 2 (*p* < 0.05) and Profile 3 (*p* < 0.001), and Profile 2 surpassing Profile 3 (*p* < 0.01). Regarding emotional support, Profile 1 again scored above Profile 2 (*p* < 0.05) and Profile 3 (*p* < 0.001), while Profile 2 maintained a significant advantage over Profile 3 (*p* < 0.01). The effect sizes were moderate to large (partial η^2^ = 0.16–0.18), underscoring the important role of emotional intelligence in the adoption and effective use of AI in academic contexts (see Table 4).

#### Correlation and Regression Between EI Profiles and AI Use Dimensions

First, it was observed that students with a high EI profile reported significantly higher scores across all three AI use dimensions: educational, informational, and emotional support. In contrast, students with a deficit in emotional repair (Profile 3) showed considerably lower levels across all dimensions, with particularly pronounced differences in educational and emotional support.

Regression analyses indicated that EI profiles explained between 20% and 29% of the variance in AI use, representing a strong and consistent association. Specifically, the emotional repair deficit predicted lower levels of AI use across all dimensions (β ranging from −0.63 to −0.80, *p* < 0.001), whereas the regulatory profile differed from the high EI profile mainly in educational and informational support, but not significantly in emotional support.

The correlations confirmed this pattern: the EI profile with a deficit in emotional repair was negatively and significantly associated with AI use across all dimensions (r ranging from −0.44 to −0.52, *p* < 0.001), indicating that as EI decreases, the diversity and purposefulness of AI use also decline (see Table 5).

These findings support the proposed hypothesis: students with higher levels of emotional intelligence tend to use AI in a more diverse and balanced manner, which promotes their well-being and academic adaptation.

## 4. Discussion

The results indicate that EI profiles differ not only in their dimensions of attention, clarity, and emotional repair (Salovey et al., 1995) but also significantly modulate the perception and utilization of AI. This provides empirical evidence supporting the interaction between emotional competencies and technological tools in higher education (López De La Cruz & Baldeón-Canchaya, 2024; Ahsqui & Martínez, 2024).

Three differentiated profiles were identified via cluster analysis: Profile 1 (High and Balanced EI), Profile 2 (Regulatory EI), and Profile 3 (Deficit in Emotional Repair). These findings align with previous research using the TMMS-24 to assess EI in university and adolescent populations (González et al., 2021; Guerra-Bustamante et al., 2019; Fernández-Berrocal et al., 2004), confirming the instrument’s utility in distinguishing functional student groups according to emotional competencies. The observed configuration of the three profiles can be explained by integrating trait and state models of emotional intelligence (EI). According to Petrides et al. (2004), trait EI reflects stable dispositions in emotional attention, clarity, and repair, which influence how individuals cope with academic stress and process emotional information in educational contexts. On the other hand, state EI, conceptualized by Mayer et al. (2016), allows emotional skills to be activated and adapted according to situational demands, such as using new technologies or managing complex academic workloads. Thus, this Profile 1, representing ~40% of the sample, reflects comprehensive emotional development with the capacity to perceive, understand, and regulate emotions effectively. This configuration supports resilience to academic stress and motivation for autonomous learning, consistent with studies highlighting EI as a predictor of academic success (Goleman, 1995; Extremera Pacheco & Fernández Berrocal, 2005). This Profile 2, characterized by moderate emotional attention but high clarity and repair, demonstrates that emotional regulation can compensate for lower attention to affective states, facilitating decision-making and management of complex academic situations (Mayer et al., 2016). This pattern aligns with findings emphasizing the role of regulation in academic resilience and autonomous learning strategies (Petrides et al., 2004; Salovey & Mayer, 1990). Finally, the Profile 3, defined by a deficit in emotional repair, shows that difficulties in regulation limit the ability to manage stress and leverage technological support resources. This finding is consistent with studies linking low repair levels with higher anxiety and lower academic performance (Extremera Pacheco & Fernández Berrocal, 2005).

Regarding the second objective, which aimed to analyze differences in the relationship between EI profiles and AI use, the results indicated statistically significant differences between groups across all three evaluated dimensions: educational, informational, and emotional support. Students in Profile 1 reported the highest levels in all dimensions, indicating that comprehensive emotional development favors both perception of technological resources and their strategic use (MacCann et al., 2020). Conversely, students in Profile 3 scored lowest, particularly in emotional support, suggesting that low regulation limits not only AI use for learning but also its potential as a socio-emotional tool (Extremera Pacheco & Fernández Berrocal, 2005).

Post hoc analyses confirmed that differences among profiles were significant across all AI dimensions, with moderate to large effect sizes. In educational support, each profile differed significantly from the others, whereas in informational and emotional support, Profiles 1 and 2 clearly differed from Profile 3. This pattern confirms that EI influences not only academic performance but also the adoption and strategic use of emerging educational technologies (Ocampo et al., 2024).

These results can be interpreted through trait and state models of emotional intelligence (EI). Trait EI (Petrides et al., 2004) reflects stable dispositions for perceiving, understanding, and regulating emotions. Students in Profile 1, with high and balanced EI, use these skills to evaluate technological resources and manage their motivation and stress when interacting with AI. In contrast, students in Profile 3, with deficits in emotional repair, lack regulatory mechanisms, which limits their cognitive and socio-emotional utilization of AI. From the perspective of state EI (Mayer et al., 2016), emotional skills are activated according to situational demands, allowing high-EI profiles to integrate AI strategically in diverse academic and socio-emotional contexts, while low emotional repair profiles face difficulties adapting to these situations.

The results can also be interpreted through technology acceptance models, namely TAM and UTAUT. In TAM (Davis, 1989), perceived usefulness and ease of use determine behavioral intention; high EI enhances these perceptions, increasing motivation to use AI in diverse and strategic ways. In UTAUT (Venkatesh et al., 2003), performance expectancy, social influence, and facilitating conditions are modulated by EI: students with high EI are more resilient, socially adaptive, and able to leverage environmental resources, thereby reinforcing AI adoption and effective use.

Post hoc analyses revealed significant differences with moderate-to-large effect sizes between profiles, particularly between Profiles 1 and 2 compared to Profile 3. This indicates that emotional regulation capacity acts as a key factor in the functional integration of emerging technologies, supporting previous findings that highlight EI as a predictor of both academic performance and strategic use of digital tools for educational and socio-emotional purposes (MacCann et al., 2020; Villafuerte, 2024).

Finally, focusing on the results for Objective 3—the relationship and regression between EI and AI use—correlational and regression analyses confirmed that EI is significantly associated with all three dimensions of perceived AI use: educational, informational, and emotional support. High-EI profiles showed strong positive correlations across all dimensions, whereas profiles with emotional repair deficits exhibited low or non-significant correlations. Multiple linear regression analyses indicated that EI, particularly the emotional repair dimension, serves as a significant predictor of strategic AI use, explaining a substantial proportion of the variance across the three dimensions.

These findings can also be interpreted through TAM and UTAUT. In TAM (Davis, 1989), technology use intention depends on perceived usefulness and ease of use; EI can enhance these perceptions, as emotionally competent students are better able to objectively evaluate AI benefits and manage the effort required for integration. Similarly, according to UTAUT (Venkatesh et al., 2003), factors such as performance expectancy, social influence, and facilitating conditions are moderated by EI: students with high EI are more resilient, more receptive to positive social influence, and better able to manage facilitating resources, which increases their intention and frequency of AI use.

Consistent with previous research, Parker et al. (2004) demonstrated that students with high EI exhibit greater academic and social adaptation, which translates into more efficient use of digital tools. Sánchez-Álvarezet al. (2020) further emphasize that EI promotes adaptive technology use, fostering self-regulation strategies, problem-solving, and stress management, aligning with the findings of the present study.

Based on these results, and from a practical perspective, findings suggest that emotional training programs can significantly improve emotional regulation, especially in students with deficits in Emotional Repair, enhancing well-being and optimizing AI use as an educational and socio-emotional tool (López-Bustamante & Vargas-D’Uniam, 2025). Additionally, technology platform design should incorporate adaptive feedback mechanisms and self-regulation resources to support students with lower emotional competence (López De La Cruz & Baldeón-Canchaya, 2024).

The results of this study should be interpreted with caution due to several methodological limitations. First, the cross-sectional design employed prevents causal inferences, meaning that it is not possible to assert that emotional intelligence (EI) directly determines the use of artificial intelligence (AI), but only that an association exists between the two variables. Additionally, the use of a convenience sample from a single university limits the generalizability of the findings to other academic and cultural contexts. Similarly, reliance on self-report measures introduces risks of social desirability bias and perceptual distortions, as students may overestimate or underestimate both their emotional competencies and their interaction with AI.

Conceptually, it is necessary to critically consider how “AI use” was operationalized. By focusing exclusively on perceptual measures, there is a risk of failing to capture the gap between perceived and actual or effective use. This has important implications, as a student may report high levels of AI utilization without this translating into real, critical, and strategic engagement with the technology.

Ethical implications also emerge that warrant attention. Promoting AI as a tool for emotional support carries the risk of overreliance on technological systems that cannot replace human support or professional mental health services. Concerns also arise regarding the privacy of sensitive data, the potential inappropriate substitution for professional care, and algorithmic biases that may reproduce inequities. To mitigate these risks, practical protective measures are recommended, such as ensuring algorithmic transparency, establishing informed consent protocols for data use, and enabling referral mechanisms to professional services in cases where AI interaction cannot replace specialized care.

Several recommendations for practice and future research stem from these limitations. First, longitudinal and multi-institutional studies are suggested to observe the evolution of AI use according to EI profiles and to compare results across different contexts. Second, it is recommended to include objective measures of technological use, such as interaction logs on digital platforms, alongside subjective evaluations, to obtain a more comprehensive and less biased perspective. Additionally, the implementation of randomized controlled trials is encouraged to rigorously evaluate the effectiveness of socio-emotional interventions linked to AI use.

From an applied perspective, the findings suggest concrete pedagogical strategies. One approach is the incorporation of emotional regulation modules into teacher training, enabling instructors to model and guide students in balanced AI use. Another recommendation is to personalize AI integration in courses based on EI metrics, adapting technological resources to the emotional needs of each student profile. A further promising strategy is training in emotional clarity, which may foster more critical and reflective AI use, reducing risks of technological dependency or the substitution of essential cognitive processes.

## 5. Conclusions

The present study’s findings indicate that emotional intelligence profiles are a determining factor in the perception and use of AI in higher education. Students with high clarity and emotional repair tend to use AI more strategically and effectively, whereas those with regulatory deficits show significant limitations, particularly in socio-emotional use. These results highlight the need to integrate emotional competence development into university education and technology platform design as a means to optimize learning, well-being, and adaptation in an increasingly digital educational context.

## Figures and Tables

**Table 1:** AI usefulness questionnaire as an academic resource.

Items	Educational	Informational	Emotional
1. AI helps me resolve important questions.	0.78	0.20	0.15
2. AI provides clear and useful information.	0.22	0.81	0.18
3. I have learned new things through AI-based assistants.	0.74	0.25	0.20
4. Interacting with AI makes me feel accompanied when I am alone.	0.15	0.18	0.72
5. AI responds in a comprehensive manner when I share something personal.	0.18	0.20	0.65
6. Interacting with AI is emotionally helpful when I feel unwell.	0.20	0.15	0.70
7. I trust the information provided by AI.	0.25	0.76	0.18
8. I feel that AI understands my questions or needs.	0.20	0.69	0.22
9. I find it easy to use AI applications or assistants.	0.35	0.45	0.20
10. I can use AI independently, without assistance from others.	0.32	0.48	0.18
11. How frequently do you use AI or virtual assistants (e.g., ChatGPT, Alexa)?	0.28	0.40	0.15
12. I am concerned that AI may provide incorrect or misleading information.	0.20	0.35	0.30
Explained Variance (%)	26	24	18

**Table 2:** Silhouette Index (K-means) and AIC and BIC Criteria (GMM).

k	Average Silhouette	AIC	BIC
2	0.568	2023.44	2096.85
3	0.482	1926.41	2038.46
4	0.386	1935.57	2086.25
5	0.327	1952.03	2141.35

**Table 3:** Descriptive Statistics of Cluster Components.

Cluster	Attention	Clarity	Repair
	M (SD)	M (SD)	M (SD)
1 (High Balanced)	6.2 (0.3)	6.0 (0.3)	6.1 (0.4)
2 (Regulatory)	5.5 (0.5)	5.2 (0.4)	5.3 (0.5)
3 (Repair Deficit)	5.0 (0.6)	4.8 (0.5)	2.9 (0.5)

**Table 4:** AI Use According to EI Profiles.

AI Dimension	Profile 1: High EI	Profile 2: Regulatory EI	Profile 3: Deficit in Repair	F_(2,349)_	*p*	η^2^ Partial
Educational support	4.20 (0.48)	3.90 (0.52)	3.50 (0.55)	40.10	<0.001	0.18
Informational support	4.10 (0.50)	3.85 (0.55)	3.45 (0.54)	36.50	<0.001	0.17
Emotional support	3.80 (0.60)	3.60 (0.62)	2.90 (0.65)	35.20	<0.001	0.16

Note: Profile 1 shows high attention, clarity, and repair with comprehensive emotional management; Profile 2 combines moderate attention with high clarity and repair, excelling in regulation and resilience; Profile 3 presents moderate attention and clarity but low repair, which hinders emotional regulation and affects motivation.

**Table 5:** Regression and Correlation Results Between EI Profiles and AI Use Dimensions.

AI Dimension	*F*(2, 349)	R^2^	Regulatory β (*p*)	Deficit β (*p*)	r
Educational Support	70.94	0.29	−0.21 (0.001)	−0.76 (<0.001)	−0.52
Informational Support	44.44	0.20	−0.18 (0.009)	−0.63 (<0.001)	−0.44
Emotional Support	57.03	0.25	−0.10 (0.196 ns)	−0.80 (<0.001)	−0.46

Note. β = regression coefficients in relation to the high EI profile (reference). r = Pearson correlation between EI level (1 = high, 2 = regulatory, 3 = deficit) and AI dimensions.

## Data Availability

The data supporting the findings of this study are available upon request from the corresponding author. Due to ethical and participant confidentiality considerations, the data cannot be openly shared publicly. The data will be provided to qualified researchers for replication or related studies, under an agreement that ensures participant privacy.
